# Resection of Large Urological Tumors With or Without Inferior Vena Cava Extension in Jehova's Witnesses

**DOI:** 10.3389/fsurg.2020.622110

**Published:** 2020-12-18

**Authors:** Gaetano Ciancio, Javier Gonzalez

**Affiliations:** ^1^Department of Surgery, University of Miami Miller School of Medicine, Jackson Memorial Hospital, Miami, FL, United States; ^2^Department of Urology, University of Miami Miller School of Medicine, Jackson Memorial Hospital, Miami, FL, United States; ^3^Miami Transplant Institute, University of Miami Miller School of Medicine, Jackson Memorial Hospital, Miami, FL, United States; ^4^Department of Urology, Hospital General Universitario Gregorio Marañón, Madrid, Spain

**Keywords:** renal cell carcinoma, adrenal carcinoma, tumor thrombus, transplant-based approach, estimated blood loss, transfusion

## Abstract

**Background:** Renal and adrenal tumors with/without tumor thrombus in the inferior vena cava (IVC) pose a challenge to the surgeon due to the potential for massive hemorrhage and tumor thromboemboli. The situation would be more critical for Jehovah's Witness (JW) patients which refuse blood transfusion. A transplant-based (TB) approach to these tumors in JWs would result a safe surgical method, providing limited blood loss and perioperative complications. We report our experience using a TB surgical approach in JW harboring large adrenal/renal tumors with/without tumor thrombus trying to determine its usefulness in this setting.

**Patients and Methods:** From 2003 to 2011, 7 patients underwent resection of renal/adrenal tumors with/without tumor thrombus in the IVC by means of a TB approach. Thrombus level was renal (*n* = 2), retrohepatic (*n* = 1), and suprahepatic (*n* = 1). The remaining 3 patients did not present thrombus. No pre-operative optimization or cell-saver were used. Estimated blood loss, perioperative complications (Clavien-Dindo and cause), hemoglobin/hematocrit loss, and length of stay were considered main outcomes.

**Results:** The intervention was successfully completed without transfusion in all cases. Operative time and blood loss were 2.5 h (range: 1.83–5.75) and 150 cc (range: 100–750), respectively. No major post-operative complications were registered. However, minor complications were detected in 57% of the patients included. Median hemoglobin loss was 1.13 mg/dL, which translated a median hematocrit loss of 2.3%. Patients were discharged in a median of 7 days (range 5–20).

**Conclusions:** A TB-surgical approach provides enhanced retroperitoneal exposure and optimal vascular control, thus limiting operative blood loss or major complication development, thus resulting useful in JWs.

## Summary

Renal and adrenal tumors with/without tumor thrombus in the inferior vena cava (IVC) pose a challenge to the surgeon due to the potential for massive hemorrhage and tumor thromboemboli. The situation would be more critical for Jehovah's Witness (JW) patients which refuse blood transfusion. The transplant based (TB) approach consists of a sequence of surgical maneuvers aimed to improve visual and vascular control in the surgical field, which in turn provide for limited blood loss and post-operative complications. We report our experience in dealing with these tumors in JW using a TB open surgical approach trying to prove its usefulness in this particular subgroup.

A total of 7 patients were included. Radical nephrectomy or adrenalectomy in conjunction with tumor thrombectomy was achieved successfully in all cases with acceptable blood loss and no transfusion requirements. No major complications were noted in the first 30 days after the procedure. An aggressive surgical approach remains the only strategy for curing patients harboring large renal/adrenal tumors, particularly for those that extend into the IVC. A TB-surgical approach may provide enhanced retroperitoneal exposure and optimal vascular control, thus limiting operative blood loss or major complication development in this subset of patients.

## Introduction

Renal and adrenal tumors infrequently extend into the inferior vena cava (IVC) (1, 2). Unfortunately, surgery remains the only potential cure for these patients (3, 4). Most surgeons would agree that this type of surgery is complex and require an excellent understanding of the anatomy (4, 5). The surgical technique has to provide adequate exposure and optimal vascular control to avoid perioperative complications, including massive hemorrhage and death.

In this context, we routinely apply the surgical principles and maneuvers derived from transplantation surgery. The transplant-based (TB) surgical approach includes the rotation of the visceral contents of the right and left of the abdomen (Mattox and Cattell-Braasch maneuvers), control of the main renal artery through a posterior plane of dissection, complete mobilization of the liver (piggy-back technique), full circumferential dissection of the IVC, and tumor thrombus safe control in its cranial end by means of relocation below the major hepatic veins entrance (milking maneuver), two-step sequential thrombus withdrawal (to maintain the natural venous drainage shunt through the liver), or abdominalization of the right atrium through the diaphragm (5). TB techniques help us to remove large renal or adrenal tumors safely, with a lower perioperative complication rate [including estimated blood loss (EBL) and transfusion requirements] even in the presence IVC tumor thrombus, and in most instances through a single abdominal approach (3, 4).

A more challenging scenario is that posed by Jehovah's witnesses (JW), given that they refuse to receive blood products or transfusions due to their religious beliefs. In these cases, we must provide for an even safer procedure if possible, by decreasing blood loss while still providing for optimal cancer control in our commitment to bring no harm to the patient. In this way, the use of TB-techniques may result useful in this unique subgroup of patients harboring large renal/adrenal tumors with/without tumor thrombus. In this report, we describe our experience managing 7 patients with renal cell carcinoma (RCC) or adrenal tumors with and without IVC tumor thrombus by means of a TB approach.

## Materials and Methods

From November 2003 to March 2011, 7 patients (3 men and 4 women) underwent resection of RCC (6 patients) and adrenal (1 patient) tumors. Four of these patients had extension of tumor thrombus into the IVC (including the patient presenting the adrenal tumor). The study was approved by the University of Miami Institutional Review Board.

Patient ages ranged from 35 to 74 years (median 58). Initial diagnosis was made by computed tomography (CT). Cardiac, renal, and respiratory status were evaluated pre-operatively. The level of the thrombus was confirmed in all patients with magnetic resonance imaging (MRI). Pre-operative embolization was not performed in any of the cases included. The cranial extent of the tumor was initially defined as per Neves and Zincke (6); however, for level III thrombi we used our modified definition (2, 7). For pathological staging the 2009-TNM classification was used. Tumor grade was classified according to the Furhman grading system. Median tumor size was 10.0 cm (range: 7.5–18.0). Given the large tumor sizes and the presence of tumor thrombus in 4 of these patients, it was decided to proceed with surgery as a first step in all cases. None of the patients had pre-operative optimization (i.e., neither erythropoietin nor iron supplementation). Informed consent was given, including specific statements regarding the complexity of the surgery, and emphasizing the risks associated with not receiving any blood products during the procedure or in the post-operative period.

The tumor was right-sided in 2, and left-sided in the remaining 5 patients. Three patients had no tumor thrombus, in two a level I tumor thrombus (i.e., renal vein only) was detected, and the remaining two patients presented level III tumor thrombi (Adrenal carcinoma + IIIa and RCC + IIIc, respectively, according to our classification) (2, 7). All patients were managed by a single transabdominal approach and without bypass maneuvers. Intraoperative transesophageal echocardiography (TEE) was used to delineate and monitor the cranial extent of the thrombus during the procedure (8) (Table 1).

**Table 1:** Main characteristics of the patients included in the series.

**Case number**	**#1**	**#2**	**#3**	**#4**	**#5**	**#6**	**#7**
**Demographic variables**							
Age (years)	62	74	48	53	58	73	35
Gender	F	M	F	M	M	F	F
Type of tumor	Renal	Renal	Renal	Renal	Renal	Renal	Adrenal
**Pre-operative diagnostic variables**							
Imaging	CT + MRI	CT	CT	CT + MRI	CT	CT + MRI	CT + MRI
Laterality	L	L	L	R	R	L	L
Tumor size (cm)	10	13	15	9	8	7.5	18
Tumor thrombus Level (N&Z)	I	–	–	III	–	I	III
Tumor thrombus level (UM)	I	–	–	IIIc	–	I	IIIa
**Operative characteristics**							
Type of surgery	RN + TT	RN	RN	RN + TT	RN	RN + TT	RN+ RA + TT
Thrombectomy	C	–	–	C	–	C	C
Surgical adjuncts	–	Extended LND	–	CircumferentialCavectomy + Extended LND	–	–	Circumferential cavectomy + Extended LND + Splenectomy
Cell-saver use	No	No	No	No	No	No	No
Bypass	No	No	No	No	No	No	No
**Intraoperative variables**							
EBL (cc)^**^	150	400	150	200	150	100	750
Operative time (min)	203	150	110	260	120	135	345
**Main outcomes**							
Pre-operative Hb (g/dL)	11	12	12	9	10.4	6.2	10
Post-operative Hb^*^ (g/dL)	10	9	10	8	10	7.5	8
ΔHb (pre-Hb-post-Hb)	1	3	2	1	0.4	−1.3	2
Pre-operative Hct (%)	32	34	31	27	31.5	21	30.5
Post-operative Hct^*^ (%)	29.4	28	29.9	25	30	24.6	24
ΔHct (post-Hct-pre-Hct)	2.6	6	1.1	2	1.5	−3.6	6.5
Post-operative Cr^*^ (mg/dL)	1.1	1.5	1.2	1.5	1.5	0.87	1.2
Transfusion rate (RBCP units)	0	0	0	0	0	0	0
Complication (30 days)	No	Yes	No	Yes	No	Yes	Yes
Clavien-Dindo category	–	II	–	II	–	II	II
Cause of complication	–	Adynamic ileus	–	Atelectasis	–	Adynamic ileus	Pleural effusion
Complication management	–	NGT	–	Respiratory Physiotherapy	–	NGT	Conservative
LOS (days)	7	6	6	12	5	12	20
pTNM 2009	pT3aN0M0	pT2bN0M0	pT2bN0M0	pT3bN1M0	pT2aN0M0	pT4NxM0	pT4N0M0
Pathology	RCC	RCC	RCC	RCC	RCC	RCC	Cortical Adrenal carcinoma
Variant	Clear cell	Clear cell	Clear cell	Papilllary	Clear cell	Clear cell + oncocytic (focal rhabdoid and sarcomatoid differentiation)	–
Fuhrman grade	II	III	III	III	III	IV	–
Follow-up (months)	3	24	27	22	36	24	18
Status at last follow-up	Lost	Alive	Alive	Alive	Alive	Alive	Death (multiple metastases)

*N&Z, Neves and Zincke Classification System; UM, University of Miami Classification System; M, male; F, Female; R, right; L, left; LOS, length of stay; RN, radical nephrectomy; TT, tumor thrombectomy; RA, radical adrenalectomy; PRBC, packed red blood cells; EBL, estimated blood loss; Hb, hemoglobin; Hct, hematocrit; Cr, creatinine; C, complete; NGT, nasogastric tube; LND, lymph node dissection; CT, computed tomography; MRI, magnetic resonance imaging*.

* *Post-operative values were determined at day post-operative 7*.

** *Estimated blood loss was measured as the amount of blood present in the vacuum device at the end of the procedure*.

Auto-donated blood was not preemptively stored. Although the use of cell saver was planned, the amount of blood loss was not significant neither during primary tumor excision, nor after IVC opening, and thus none of the patients received autologous blood from this device.

### Operative Technique

The TB-technique has already been described at length, for both right and left renal or adrenal tumors (9, 10). Briefly, a sub-costal incision was made commencing ~2 fingerbreadths below the right or left costal margin (according the tumor location), extending out laterally to the mid axillary line. A Rochard self-retaining retractor was placed, elevating the costal margins and splaying them laterally toward the axillae.

**Figure 1:**
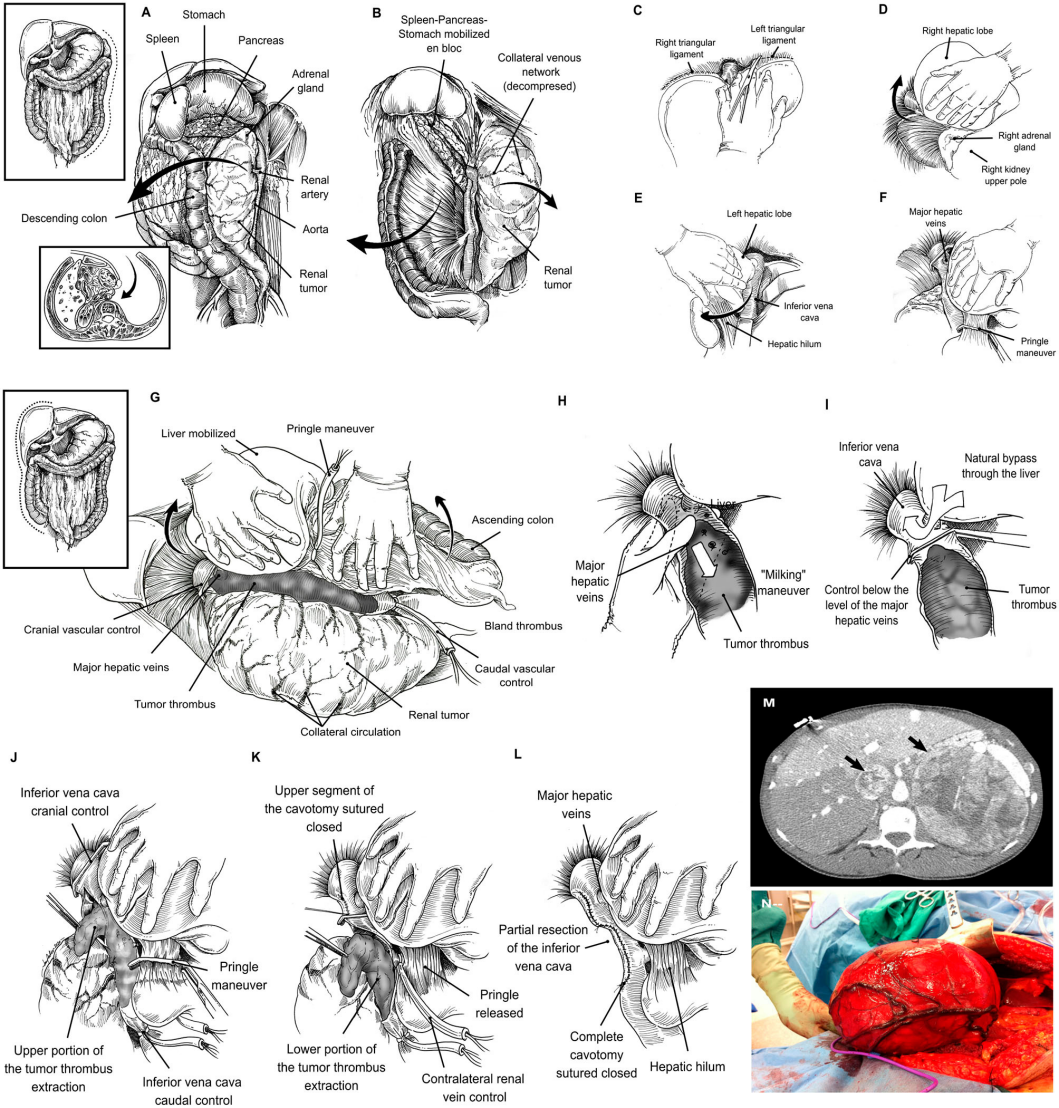
Left-sided tumors required the mobilization of the spleen, pancreas and stomach en bloc. In addition, the left adrenal gland and kidney along with its envelopes are mobilized toward the midline to gain access to the main renal artery **(A)**. Once the main renal artery is divided, an anterior plane of dissection is developed by separating the descending colon from the anterior aspect of the kidney **(B)**. Liver mobilization: The round and falciform ligaments are divided, and the incision is carried upwards around both the right superior coronary and triangular ligaments, and by passing to the left side, to the left triangular ligament **(C)**. The visceral peritoneum on the right of the hepatic hilum and the infrahepatic IVC is incised in conjunction with the right triangular and hepato-renal ligaments and the liver is gradually rolled to the left. By doing so, adequate exposure of the right upper abdominal quadrant is gained, thus facilitating the removal of large masses located in the kidney upper pole or the adrenal gland **(D)**. The division of the left triangular ligament allows the mobilization of the left lobe of the liver to the midline **(E)**. The liver is detached from the anterior aspect of the inferior vena cava until it lies in a “piggy-back” fashion. By means of Pringle maneuver and suprahepatic caval control, the liver circulation is completely interrupted **(F)**. Right visceral mobilization allows excellent exposure of the infrahepatic, intrahepatic, and suprahepatic portions of the IVC **(G)**. “Milking” maneuver: The thrombus is “milked” to a level below the ostium of the MHVs **(H)**, and the IVC is cross-clamped between this level and the tumor thrombus cranial end **(I)**. Cavotomy in a two-step fashion: Hepatic vascular exclusion is performed by means of Pringle maneuver and suprahepatic IVC control. The infrarenal IVC, and contralateral have also been controlled. Once the hemodynamic stability is warranted, a first part of the cavotomy is carried out between the level of the MHVs to the right adrenal vein, and the tumor thrombus is sharply dissected-off and removed from this IVC segment **(J)**. Thereafter, the upper segment of the IVC is sutured closed and, the Satinsky clamp is repositioned below the MHVs, Pringle is released, and normal liver blood flow reestablished. The cavotomy is now extended in caudal direction until the inferior border of the renal vein is reached. The tumor thrombus is removed from the caval lumen by means of a mix of blunt and sharp dissection **(K)**. A variable portion of IVC wall surrounding the renal vein ostium is excised with the specimen. Finally, the IVC lumen is flushed with heparin solution, and the lower segment of the cavotomy is sutured closed **(L)**. Large adrenal tumor with tumor thrombus (black arrows), the lack of space due to the size of the tumor may be a major problem **(M)**. Large right renal tumor without tumor thrombus. By mobilizing the abdominal upper quadrants exposure of the retroperitoneal space is enhanced, thus facilitating the access to the renal artery thereafter **(N)**.

We pursued early intraoperative ligation of the involved renal artery. The kidney was mobilized medially with the liver, or en bloc with the spleen and pancreas, until the renal artery was identified, ligated, and divided (11) (Figure 1). Arterial ligation resulted in a rapid decompression of the collateral network venous circulation, thus favoring a decrease in blood loss, and in turn preventing the need for blood transfusions.

Exposure of the left kidney was facilitated by mobilizing the descending colon, and the spleen en bloc with the pancreas toward the midline, thus gaining access to the entire retroperitoneal space (Figures 1A,B). Similarly, the exposure in the right side required liver mobilization. For this purpose, the dissection began with the division of the round and falciform ligaments with cautery, and this incision carried upwards around both the right superior coronary ligament, and by passing to the left side, dividing the left triangular ligament. The visceral peritoneum on the right of the hepatic hilum and the infrahepatic IVC was incised in conjunction with the right inferior coronary and hepato-renal ligaments. The liver can be then gradually rolled to the left (9). By doing so, adequate exposure of the right upper abdominal quadrant is gained, thus facilitating the removal of large masses located in the kidney upper pole or the adrenal gland (12) (Figures 1C–F). Thereafter, the liver was dissected-off the IVC by dividing the minor hepatic veins crossing from the right and caudate lobes to its anterior aspect, until the liver lies in a “piggyback” fashion; completely detached from the IVC, except for the major hepatic veins(MHVs) (1). In this manner, the infrahepatic, intrahepatic, and suprahepatic portions of the IVC are fully exposed (Figure 1G). In addition, a plane of dissection between the IVC and the posterior abdominal wall was developed by the identification, ligation and division of the small tributaries located at this level that may become engorged to look like lumbar vessels. This step is crucial, to obtain complete circumferential vascular control of the IVC. Additionaly, in the presence of level III tumor thrombus, an opening in the lesser omentum allowed the porta hepatis to be controlled with a Rummel tourniquet; a Pringle maneuver can then be carried out (i.e., temporarily occluding the portal venous and arterial inflow to the liver) as required.

A useful technique, which we have applied for a thrombus located at or above the MHVs, (levels IIIb-IV) is to “milk” the thrombus to a level below their ostium and cross-clamp the IVC just between this level and the tumor thrombus cranial end (1) (Figures 1H,I). The “milking” maneuver is often feasible to perform, since ligation of the renal artery reduces the blood supply to the tumor thrombus, thus generating a loss of turgidity and consistency thereof and permitting certain degree of additional compressibility in caudal direction. This maneuver serves a dual function. First, it allows the liver to drain into the IVC avoiding hypotension from decreased venous return (pre-load). Second, by not clamping the MHVs or porta hepatis, liver congestion and post-operative hepatic dysfunction are avoided. Nevertheless, the surgeon must be careful when manipulating the thrombus so as to avoid dislodging it.

In cases in which the “milking” maneuver is not possible, we recommend the performance of the Pringle maneuver to temporary occlude the inflow to the liver. The infrarenal IVC, and contralateral renal/adrenal veins have to be controlled sequentially thereafter, and a trial with Satinsky clamp placed across the suprahepatic IVC is made. At this moment the patient is checked for hemodynamic stability. If the patient remains stable, then the collateral venous return is noted to maintain enough cardiac pre-load and the surgeon may proceed with the cavotomy (Figures 1K,L). Otherwise a rapid infuser should be used to maintain the cardiac pre-load with sustained crystalloid infusion through a central line.

Once the hemodynamic stability is warranted, a first part of the cavotomy is carried out between the level of the MHVs to the right adrenal vein, and the tumor thrombus is sharply dissected-off and removed from this IVC segment. The interior aspect of the three MHVs can be directly visualized at this moment, their orifices inspected, and thrombus located within their lumen also removed. Thereafter, the upper segment of the IVC is sutured closed, and the Satinsky clamp is repositioned below the MHVs, Pringle is released, and normal liver blood flow reestablished (1–5). The cavotomy is now extended in caudal direction until the inferior border of the renal vein is reached. The tumor thrombus is removed from the caval lumen by means of a mix of blunt/sharp dissection. A variable portion of IVC wall surrounding the renal vein ostium is excised with the specimen. Finally, the IVC lumen is flushed with heparin solution, and the lower segment of the cavotomy is sutured closed (7, 9).

In those tumors without tumor thrombus, the lack of space due to the size of the tumor was the major problem (Figures 1M,N). The abdominal upper quadrant mobilization (right or left according to tumor laterality) was carried out (10, 12) as a first step to increase exposure of the retroperitoneal space, thus facilitating the access to the renal artery thereafter (11). Once the renal artery was ligated and divided, the surgery was easier to perform, and almost no blood loss noted. Evicel® was used over the remnant of the renal hilum, renal fossa, and along the cavotomy of the IVC.

### Statistical Methods

Main outcome variables considered were: post-operative complications occurred in the first 30-day post-operative period (i.e., severity according to the Clavien-Dindo classification system and cause), estimated blood loss (EBL) during the procedure (measured as the amount of blood present in the vacuum device at the end of the procedure), pre-operative and post-operative hemoglobin (Hb) and hematocrit (Hct) levels (determined in the pre-operative analysis and blood test panel performed at post-operative day 7) and their respective delta values (i.e., Hb and Hct loss), and length of hospital stay. Numerical variables were expressed as medians and ranges, while categorical variables were expressed as frequencies and percentages.

## Results

Diagnostic information, operative characteristics, and surgical outcomes of the patients included in the series are provided in Table 1.

All RCC patients underwent radical nephrectomy, while adrenalectomy in conjunction with nephrectomy and splenectomy was required in the patient harboring the adrenal tumor, in order to achieve a complete surgical excision. All patients underwent hilar lymph node dissection, but in three of them (patients #2, #4, and #7) the lymph node dissection was extended including the para-aortic (left side), para-caval (right side), inter aortocaval, or those palpable nodes noted, depending on the primary tumor location and the particular situation of the surgical field.

Four out of seven patients (57%) required also tumor thrombectomy. In these cases, the cranial end of the tumor thrombus correlated well between the MRI depiction and operative findings (2). Tumor thrombus removal was complete in all cases. Level I tumor thrombi (patients #1 and #6) required tangential IVC wall resection en-bloc with the tumor thrombus, and primary closure of the cavotomy after thrombus removal. Stapled circumferential cavectomy between the distal limit of the major hepatic veins ostia and the caval bifurcation was performed in cases harboring a level III tumor thrombi (patients #4 and #7). While the level IIIa tumor thrombus did not require additional maneuvers, stapling of the IVC's cranial end in the case showing a level IIIc tumor thrombus was facilitated by the performance of a sequential two-step cavotomy. Cardiopulmonary or veno-venous bypass were not required. Median operative time was 2.5 h (range: 1.83–5.75) and was sensibly longer for those cases presenting tumor thrombus (particularly level III) and/or neighboring visceral compromise.

Pulmonary embolism was not detected neither intraoperatively, nor in the post-operative period. EBL ranged from 100 to 750 cc (median 150 cc). Pre-operative and post-operative values for hemoglobin and hematocrit, and their respective delta values are shown in Table 1. All the patients had a degree of anemia before surgery, which worsened shortly after surgery despite minimizing intra-operative fluids, use of albumin, and using pediatric tube phlebotomy [Median ΔHb (pre-Hb-post-Hb): 1.13 mg/dL; Median ΔHct (pre-Hct-post-Hct): 2.3%]. Of note, patient #6 presented with severe gross hematuria at the debut. She was scheduled for immediate emergency surgery, thus making pre-operative optimization impossible due to the poor pre-operative situation. The intervention provoked hematuria cessation, and hemodynamic stabilization. The post-operative analysis was performed on post-operative day 7 to limit additional blood loss, given that there were no clinical signs of sustained hemorrhage that forced us to act differently. By then, the adynamic ileus had recovered, hydration optimized, and intravenous iron supplements initiated, thus compensating the initial hemoglobin level.

Each of the 7 patients had an uneventful post-operative course. Four patients (57%) developed minor complications (Clavien-Dindo I-II). Two patients developed ileus (patients #2 and #6) that resolved with the placement of a nasogastric tube. One patient showed atelectasis in the immediate post-operative period (patient #4) that was managed conservatively with respiratory physiotherapy, and the single patient with an adrenal tumor developed bilateral pleural effusions (patient #7); however, she did not require drainage. No major complications (Clavien-Dindo III-V) were registered. Median length of hospital stay (LOS) was 7 days (range 5–20).

## Discussion

The surgical procedure for patients presenting with large renal or adrenal tumors is high-risk, complex, and challenging due to the association of such tumors with difficult-to-reach exposures, multiple venous collaterals, risk of developing pulmonary emboli, and major perioperative blood loss. The degree of complexity and the inherent risks are higher when the IVC is compromised by tumor extension, and even worse for JWs patients. In this subset, the risk is particularly sharp regarding perioperative blood loss, because these patients will not accept transfusions of any blood products based on their religious beliefs (13). In fact, West (13) mentioned that, “if the pre-operative clinical status of the patient will mandate the use of blood products during organ transplantation, then a JW patient should probably not be a candidate if he or she would refuse such transfusions.”

Unfortunately, surgery is the only viable option that exists today for attempting a cure in any patient harboring one of the above mentioned complex urological tumors. Regarding the need to perform any major abdominal or thoracic surgery in JW patients, there are few citations in the literature describing the surgical procedures applied with relative success in this patient population using multidisciplinary management and successful blood conservative techniques (14–22). The experience in treating JW patients having a renal or adrenal tumor with or without tumor thrombus extending into the IVC is scarce, and most of the literature is the form of case reports (14–18). To the best of our knowledge, the current study is the largest series reported to date of JW patients harboring large renal or adrenal malignancies extending or not into the IVC that were resected safely, in an almost bloodless field (due to early ligation of the renal and adrenal arteries), and without the use of blood products.

When dealing with a renal or adrenal tumor that also includes tumor thrombus, the critical part of the operation is management of the IVC (1–3). The important goals are to minimize bleeding and prevent the development of pulmonary emboli from the thrombus during surgery, given that either of both events can lead to fatal consequences. These points are even more important when dealing with JW patients presenting these entities. The safety net of transfusion of blood products, or use an emergency cardiopulmonary bypass would not represent an option.

Over the years, we have developed several approaches to aid in the safe removal of these tumors. The improvement in surgical technique has often been due to the application of surgical principles from different disciplines. We blended lessons learned in the fields of transplant surgery and urologic oncology to deal with large renal/adrenal tumors eventually extending into the IVC. The concept of resorting to an entirely intra-abdominal approach without cardiopulmonary or veno-venous bypass is the byproduct of this approach (1–3, 5, 9, 10). In opposition to bypass maneuvers, the TB approach helps in facilitating the resection of these tumors by increasing the exposure at the retroperitoneal space.

We have never pre-operatively embolized any of our patients (1, 2, 5), but an important principle of our surgical approach includes mobilization of the kidney with early ligation of the renal artery. The kidney mobilization begins laterally and proceeds posteriorly paying special attention to control perirenal collateral circulation. With the posterior approach, fewer varices are encountered as opposed to dissection anterior to the kidney. Once the kidney is mobilized medially, the renal artery is identified, ligated and divided, thus favoring a rapid collapse of the collateral circulation, and in turn making the rest of the dissection easier. This maneuver aims to provide the same “devascularization effect” of pre-operative embolization but avoiding its known morbidity risks (11).

Minimally invasive approaches have been gaining acceptance in the treatment of RCC with caval extension in the last decade. Experience with these approaches is still limited by the low volume of the series reported (particularly for level III-IV tumor thrombi) although perioperative outcomes seem to be promising. Nevertheless, future research is needed to prove its non-inferiority as compared to open surgery and to define its benefits and limits (23, 24). In the meanwhile, there is a trend to replicate the lessons learned from the open approach in the minimally invasive field. In fact, an example of this adaptation has been recently reported by Shen et al. (25). A modification of the “milking” maneuver was used to successfully remove 12 cases of RCC in conjunction with level III-IV tumor thrombi by means of a robotic approach, thus reinforcing that the concepts provided here will be probably still valid in the next future (26–30).

Our experience here reported, reaffirms the safe use of an organ TB approach for resection of large RCC and adrenal tumors, even in conjunction with tumor thrombus extending into the IVC in a unique, and particularly challenging subset of patients. However, our study is not without limitations. This is a small retrospective series, and the outcomes reported here have to be evaluated accordingly. It also represents the experience of a high-volume single surgeon from a referral center. Therefore, the experience with this approach may not be similar for other surgeons with less experience or other practice settings.

## Conclusions

A surgical TB approach to large renal or adrenal tumors extending or not into the IVC may provide the surgeon for an excellent exposure of the retroperitoneal space and optimal vascular control, thus facilitating minimal blood loss, and development of perioperative complications, especially in JW patients. Early ligation of the renal artery helps with decreasing blood loss by collapsing the collateral circulation, while avoiding the need of renal artery embolization and its inherent risks.

## Data Availability Statement

The raw data supporting the conclusions of this article will be made available by the authors, without undue reservation.

## Ethics Statement

The studies involving human participants were reviewed and approved by The Miami Transplant Institute Review Board approved the current study. The patients/participants provided their written informed consent to participate in this study.

## Author Contributions

GC: project development, data collection and management, data analysis, manuscript writing and editing, and final approval. JG: project development, manuscript writing and editing, figures, and final approval. All authors contributed to the article and approved the submitted version.

## Conflict of Interest

The authors declare that the research was conducted in the absence of any commercial or financial relationships that could be construed as a potential conflict of interest.
